# Enhanced electrochemical performance of nanoplate nickel cobaltite (NiCo_2_O_4_) supercapacitor applications†

**DOI:** 10.1039/c8ra09081e

**Published:** 2019-01-09

**Authors:** Anil Kumar Yedluri, Hee-Je Kim

**Affiliations:** School of Electrical Engineering, Pusan National University Busandaehak-ro 63beon-gil, Geumjeong-gu Busan 46241 Republic of Korea yedluri.anil@gmail.com yedluri.anil@pusan.ac.kr +82 51 513 0212 +82 10 3054 8401

## Abstract

Well-ordered, unique interconnected nanostructured binary metal oxides with lightweight, free-standing, and highly flexible nickel foam substrate electrodes have attracted tremendous research attention for high performance supercapacitor applications owing to the combination of the improved electrical conductivity and highly efficient electron and ion transport channels. In this study, a unique interconnected nanoplate-like nickel cobaltite (NiCo_2_O_4_) nanostructure was synthesized on highly conductive nickel foam and its use as a binder-free material in energy storage applications was assessed. The nanoplate-like NiCo_2_O_4_ nanostructure electrode was prepared by a simple chemical bath deposition method under optimized conditions. The NiCo_2_O_4_ electrode delivered an outstanding specific capacitance of 2791 F g^−1^ at a current density of 5 A g^−1^ in a KOH electrolyte in a three-electrode system as well as outstanding cycling stability with 99.1% retention after 3000 cycles at a current density of 7 A g^−1^. The as-synthesized NiCo_2_O_4_ electrode had a maximum energy density of 63.8 W h kg^−1^ and exhibited an outstanding high power density of approximately 654 W h kg^−1^. This paper reports a simple and cost-effective process for the synthesis of flexible high performance devices that may inspire new ideas for energy storage applications.

## Abstract 2

Schematic representation of the synthesis of nanoplate-like NiCo_2_O_4_ structure on nickel foam (NF).

## Graphical abstract

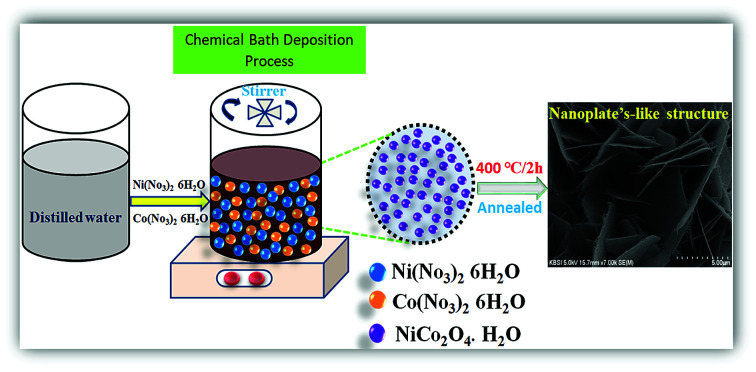

## Introduction

The development of renewable energy conversion systems is an urgent requirement to meet the needs of high power electronic devices, electric vehicles, and electrochemical energy storage devices as well as address ecological concerns.^1–3^ Among the various electrochemical energy storage systems, supercapacitors have attracted increasing attention in recent years because they exhibit a good balance between energy density and power density, excellent long-life cycling stability with a rapid charge/discharge rate over batteries, and store more energy than conventional capacitors.^4–9^

Electrode materials can be classified into two main groups based on the charge storage mechanism: electric double-layer capacitors (EDLCs) and pseudocapacitors (PCs).^10,11^ Generally, EDLCs consist of conductive porous carbon materials, such as carbon spheres, activated carbon, and graphene.^12–16^ On the other hand, the practical applications of EDLCs have been limited by their low specific capacitance and low energy density. The main pseudocapacitive materials, such as metal oxides, hydroxides and conducting polymers, exhibit higher specific capacitance and energy densities through rapid, reversible, multi-electron, surface faradaic reactions.^17–21^ In particular, some low-cost transition metal oxides and hydroxides, such as ZnO, Fe_2_O_3_, MnO_2_, Mn_3_O_4_ Co_3_O_4_, NiO, and Co(OH)_2_, have been developed as candidates.^22–26^

Among the various electroactive materials reported thus far, nickel oxide (NiO) is a typical PC material owing to its strong theoretical capacity, excellent reversibility, well-maintained and fascinating morphology, suitable pore size, large specific area, and excellent reliability.^27–29^ However, NiO as an active material in PCs has been restricted because of its lower rate behavior, poor cycling stability, and lower electrochemical activity.^30–32^ To overcome this problem, further efforts have been made to prepare nanocomposites that combine NiO with other electroactive materials. Among the various PCs components, cobalt oxides (Co_3_O_4_ and CoO) have become promising candidates for supercapacitor electrode applications owing to their low toxicity, low parity cost, facile preparation, and good corrosion stability.^33,34^ Moreover, binary metal oxides exhibit better electrochemical performance compared to single-component metal oxides owing to their outstanding electrical conductivity and multiple oxide states.

In particular, ternary nickel cobaltite (NiCo_2_O_4_) has attracted considerable attention for its ultrahigh specific capacitance because ternary NiCo_2_O_4_ combines the characteristics of simple transition metal oxides, has high specific capacitance and electronic conductivity, and better electron transport between the electrolyte and electrode surface area than binary metal oxides of nickel oxide (NiO) and cobalt oxide (Co_3_O_4_).^35,36^ In particular, NiCo_2_O_4_ has attracted considerable attention, owing to its superior conductivity to other binary metal oxides, such as ZnFe_2_O_4_, CoMoO_4_, MnMoO_4_, Zn_3_V_2_O_8_, and NiMnO_3_. Nickel cobaltite (NiCo_2_O_4_) has excellent redox activity, natural abundance, low cost, environmentally benign characteristics, and nontoxicity.^37,38^ Thus, the fabrication of nanoplate-structured NiCo_2_O_4_ using a simple and novel method to enhance its electrochemical performance is the focus of this research. Until now, there are only a few reports on the use of NiCo_2_O_4_ as an electrode material for supercapacitor applications. The chemical bath deposition approach is a more common method for the preparation of functional materials than hydrothermal methods, microwave irradiation method, thermal annealing treatment approach, and mechano-chemical synthesis. Despite this, little attention has been paid to the synthesis of NiCo_2_O_4_ electrode materials. The production of high-performance supercapacitors for nanostructured NiCo_2_O_4_ materials by a simple method remains a challenge.

In this study, a nanostructure of NiCo_2_O_4_ nanoplate-like structure was grown uniformly on highly conductive nickel foam for high-performance supercapacitor applications through a chemical bath deposition method. By rationally controlling the growth kinetics of NiCo_2_O_4_, the electrode exhibited a honeycomb nanostructure on a nickel foam surface, which provided rapid channels for ion and electron transfer and formed abundant electrochemically active sites exposed to the electrolyte surface area. The NiCo_2_O_4_ nanoplate's electrode exhibited a high specific capacitance of 2791 F g^−1^ at a current density of 5 A g^−1^ and delivered an excellent energy density 63.8 W h kg^−1^ and high power density of 654 W kg^−1^ in a KOH electrolyte. This might be because the unique interconnected nanoplate's structure exhibits good reversibility and lower charge-transfer resistance of 0.2 Ω during the faradaic process. These supercapacitors also exhibited excellent long-life cycling stability of approximately 99.1% retention after 3000 cycles, which might be because the nanoplate nanostructure allows convenient ion transport within and between the combs.

## Experimental section

### Preparation of the NiCo_2_O_4_ nanoplate material on nickel foam

All chemicals applied, such as nickel nitrate hexahydrate, cobalt nitrate hexahydrate, urea, and ammonium fluoride, were of analytical grade and used as received. The nanoplate-like NiCo_2_O_4_ nanostructure was synthesized by a simple chemical bath deposition method. The process involved a solvothermal reaction of nickel and cobalt ions as inorganic components with urea and ammonium fluoride to form a precursor/self-sacrificing template, which was followed by heat treatment in air to generate nanoplate-like structures. Prior to synthesis, a piece of nickel (1 cm × 1 cm) foam was cleaned by being immersed in 2 M HCl under ultrasonication in sequence for 15 min, and rinsed with absolute ethanol and deionized (DI) water, respectively, which results in the removal of the surface layer and greasing of Ni oxide. In particular, an aqueous homogeneous solution of all chemicals applied, such as 0.42 g of Ni (NO_3_)_2_·6H_2_O and 0.86 g of Co(NO_2_)_3_·6H_2_O, 0.22 g of CH_4_N_2_O and 1.80 g of urea were dissolved in 100 mL of deionized water with constant magnetic stirring for 50 min. After stirring, the homogeneous solution formed a clear pink color solution, which indicates the active electrode of nickel cobaltite (NiCo_2_O_4_) material. The resulting clear solution containing a piece of nickel foam was transferred into a 100 mL capped bottle and kept at 120 °C for 12 h. After cooling to room temperature, the as-prepared nickel foam was separated by centrifugation and rinsed with absolute ethanol and distilled water. The as-prepared product was then dried with a dryer for 30 min. Finally, for the heat treatment step, the resulting prepared product was annealed under 400 °C for 3 h at a heating rate of 20 °C min^−1^ in air. The as-prepared samples supported on nickel foam were obtained. The total average NiCo_2_O_4_ loading was 3 mg cm^−2^.

### Material characterization

**Scheme 1:**
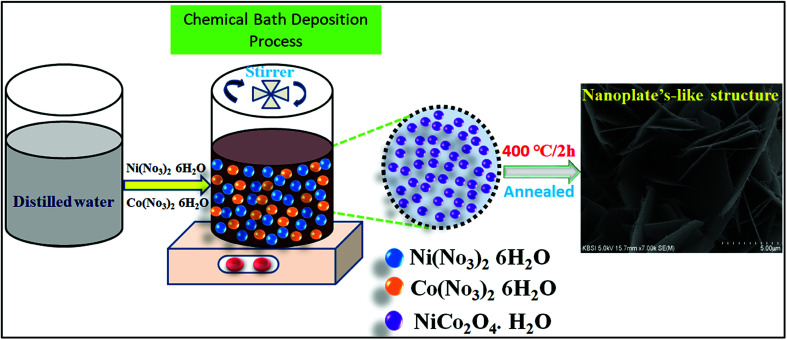
Schematic of the preparation process of NiCo_2_O_4_ nanoplate's structure.

The morphology, microstructure, and internal structural properties of the samples were studied by field emission scanning electron microscopy (FESEM, S-4800, Hitachi) at an acceleration voltage of 15 kV and field emission transmission electron microscopy (FETEM, JEOL, JEM-2100F) operated at 200 kV. The crystalline phase structure of the as-prepared sample was investigated by powder X-ray diffraction (XRD, D8 ADVANCE Bruker) at a voltage of 40 kV and a current of 40 mA using Cu Kα radiation (*k* = 0.1542 nm). The elemental composition of the material was determined by X-ray photoelectron spectroscopy (XPS, VG Scientific ESCALAB 250) using Al Kα radiation (Scheme 1).

### Electrochemical measurements

Electrochemical characterization of the NiCo_2_O_4_ were carried out in a 3 M aqueous KOH solution on an electrochemical workstation (BioLogic-SP150 Korea) using a conventional three electrode system. A three-electrode system containing Ag/AgCl and platinum foil (Pt) as the reference and counter electrode, respectively, was used. The as-prepared NiCo_2_O_4_ electrode were directly used as the working electrode. Cyclic voltammetry (CV) of the resulting product was performed over the potential range from −0.2 to 0.5 V *vs.* Ag/AgCl at different scan rates. The galvanostatic charge–discharge curves test of the prepared electrode was performed over the potential range, 0 to 0.4 V *vs.* Ag/AgCl, at various current densities (A). Electrochemical impedance spectroscopy (EIS) was conducted over the frequency range, 0.1 Hz to 100 kHz with an AC amplitude of 5 mV. The specific capacitance (*C*_sc_, F g^−1^) of the electrode from GCD plots, energy density (*E*, W h kg^−1^) and power density (*P*, W kg^−1^) for the three electrode system were calculated using the following equations (eqn (1), (2), and (3)) given below:^39^1
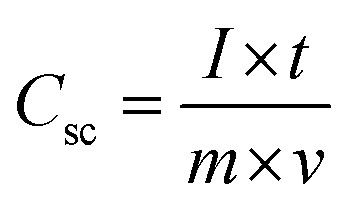
2
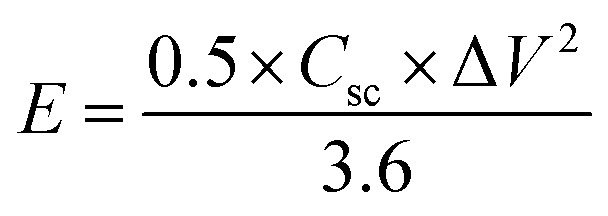
3
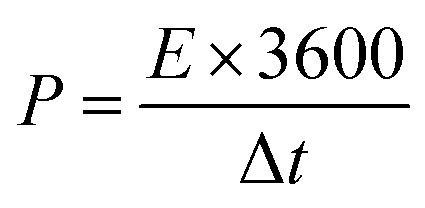
where *I* (A) is the discharge current, Δ*t* (s) is the discharge time, *m* (g) is the mass of the electroactive material of the active electrode, and Δ*V* (V) is the voltage window drop, respectively.

## Results and discussion

**Fig. 1:**
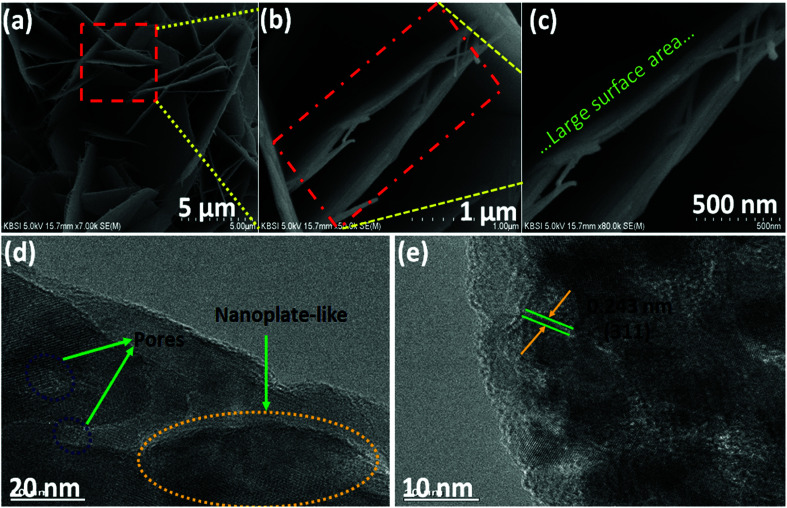
(a–c) Low- and high-magnification FE-SEM images and (d and e) TEM and HR-TEM images of the NiCo_2_O_4_ nanoplate material.

The morphology, interior structure, and crystalline properties of the as-prepared NiCo_2_O_4_ nanomaterial was examined by SEM, TEM and high-resolution TEM (HR-TEM), as shown in Fig. 1. Fig. 1a–c shows the morphology of the nanoplate-like NiCo_2_O_4_ at various magnifications. The nickel foam is covered uniformly by NiCo_2_O_4_ nanoplate structures, indicating that the nanoplates are composed of many nanosheets. The fine nanoplates had a mean diameter of 50–80 nm. As shown in Fig. 1c, the internal structure of the as-prepared material has larger pores because of the empty spaces between the plates. In particular, this nanostructure has high specific surface areas with a bimodal pore size distributions, which play a key role in the high energy density and power density suitable for energy storage applications. Moreover, FE-SEM images of the working electrode showed that every nanoplate had grown uniformly on the nickel foam and had a rough surface area. The SEM image (Fig. S1a†) of the NiCo_2_O_4_ at low magnification shows that a layer of precursor is evenly covered on the surface of Ni foam. After the nanoplate's growth, the NiCo_2_O_4_ nanoplates covered the surface of nickel foam uniformly and also nanoplates grown on nickel foam were uniformly distributed with no overlap. The magnified image in the inset reveals shows a low magnification SEM image of the visible networks of Ni foam, in which we can see that the Ni foam has a porous network structure with smooth surface and also no impurities. Fig. 1d and e presents TEM and HR-TEM images taken on the surface of the NiCo_2_O_4_ nanoplates coated on the surface of nickel foam. As shown in Fig. 1d, the internal structure of the active material clearly showed large pores and nanoplate regions. Atomic force microscopy (AFM) is a promising technique for studying some structural information and thickness measurements about the as-obtained material. Fig. S2a and b† shows topology of the surface of NiCo_2_O_4_ on Ni foil. It can be seen that a plate structure of NiCo_2_O_4_ is formed on Ni foil. The AFM images reveal a uniform surface roughness, a smooth surface, and good adhesion to the substrate with a narrow particle size distribution. Also, it can be observed that the surface is converted to a relatively uniform size. Furthermore, AFM measurement indicated that the average thickness of these NiCo_2_O_4_ nanoplate was around ∼20 to 30 nm. Overall, AFM indicates that the NiCo_2_O_4_ contains more electrocatalytic activity sites for the reaction of the redox couple in the KOH electrolyte and has a larger surface area between the electrode and electrolyte. The lattice fringes in the HR-TEM image separated by *d* = 0.243 nm were assigned to the (311) plane of NiCo_2_O_4_ (JCPDS) data, as shown in Fig. 1e.

**Fig. 2:**
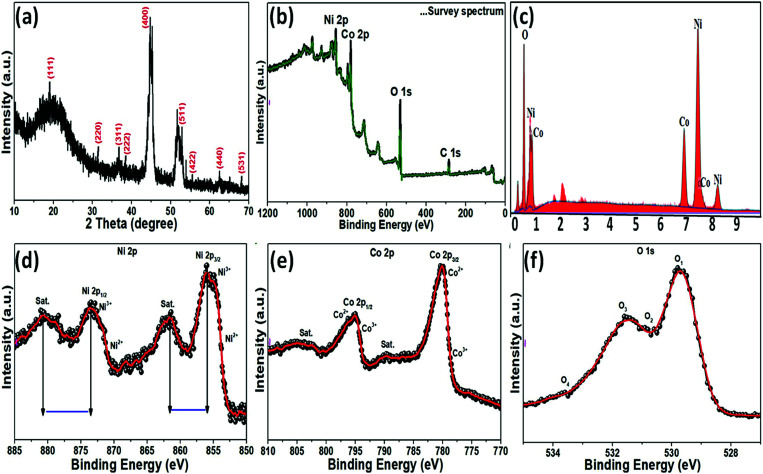
Spectroscopic and microscopic characterization of the nanoplate-like NiCo_2_O_4_ nanostructure. (a) XRD pattern. (b) XPS survey spectrum. (c) EDX spectrum and high-resolution XP spectra of (d) Ni 2p, (e) Co 2p and (f) O 1s for the as-prepared NiCo_2_O_4_ sample.

The crystallinity and phase composition of the as-prepared NiCo_2_O_4_ nanoplate on nickel foam were examined by XRD, as shown in Fig. 2a. XRD showed that the NiCo_2_O_4_ material was crystalline. The XRD peaks at 18.91°, 31.15°, 36.70°, 38.40°, 44.62°, 55.44°, 59.09°, 64.98°, and 68.31° 2*θ* were indexed to the (111), (220), (311), (222), (400), (422), (511), (440), and (531) planes, respectively, of the cubic spinal-like NiCo_2_O_4_ Joint Committee in Powder Diffraction Standards (JCPDS card no. 20-0781),^40^ which is consistent the TEM analysis. XPS provided further evidence of the chemical composition and elemental oxidation states of the as-prepared NiCo_2_O_4_ sample. The XPS survey spectrum revealed nickel, cobalt, and oxygen in accordance with the literature values for NiCo_2_O_4_ (Fig. 2b). EDX analysis of the as-prepared product showed nickel, cobalt and oxygen (Fig. 2b), indicating a pure phase without impurities. The atomic percentage of Ni, Co, and O were 41.87%, 35.39%, and 22.73% (5 : 4 : 3), respectively. A Gaussian fit of Ni 2p deconvoluted spectrum at 855.8, 861.8, 873.5, and 879.9 eV revealed Ni^2+^ 2p_3/2_, Ni^3+^ 2p_3/2_, Ni^2+^ 2p_1/2_ and Ni^3+^ 2p_1/2_, respectively (Fig. 2d), indicating the presence of two spin–orbit doublets and two shakeup satellites denoted as (“Sat.”) as well as +2 and +3 oxidation states in the prepared NiCo_2_O_4_ material.^41^ In the case of the Co 2p (Fig. 2e) deconvoluted spectrum, multiple peaks at 780.5, 789.6, 795.1 and 804.2 eV were assigned to Co^2+^ 2p_3/2_, Co^2+^ 2p_3/2_, Co^3+^ 2p_1/2_, and Co^2+^ 2p_1/2_, respectively.^42^ The high resolution O 1s spectrum (Fig. 2f) was deconvoluted into two main peaks, 530.2 and 530.9 eV, which confirmed the formation of metal–oxygen bonding in the presence of O 1s.^43^ The data clearly show that the presence of valence and mixed valence of metal ions, Ni^2+^/Ni^3+^, Co^2+^/Co^3+^, and O 1s, on the surface of the as-prepared NiCo_2_O_4_ sample, which is expected to provide sufficient active sites for the redox reaction in the electroactive activity.

**Fig. 3:**
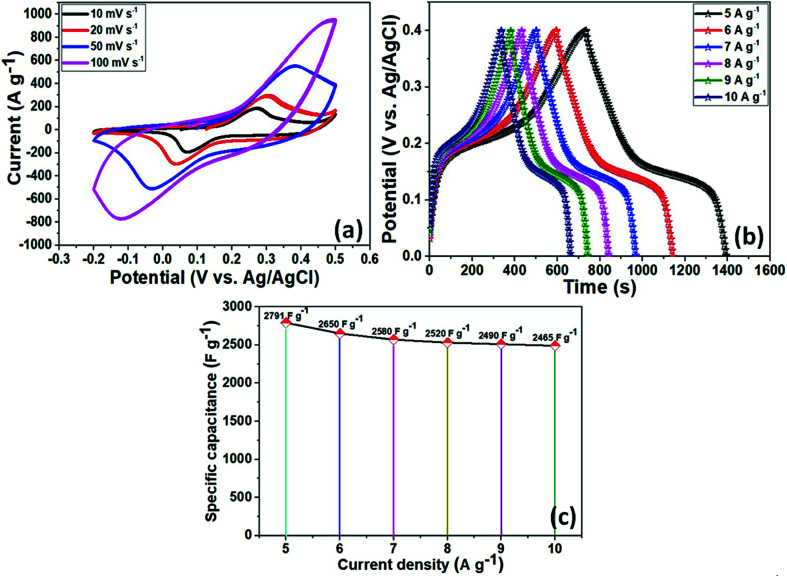
(a) CV curves of the NiCo_2_O_4_ nanoplate's obtained at various scan rates of 10–100 mV s^−1^; (b) galvanostatic charge–discharge curves of the NiCo_2_O_4_ at different current densities of 5–10 A g^−1^; (c) specific capacitance of the three electrode material at various current densities.

The electrochemical performance of as-prepared NiCo_2_O_4_ as an electrode material for supercapacitors was investigated using a three-electrode system in a 3 M KOH aqueous solution as the electrolyte. Fig. 3a presents the CV curves of the active electrode. All CV curves showed a pair of redox peaks at various scan rates from 10–100 mV s^−1^ over the voltage range of −0.2 to 0.5 V, indicating similar pseudocapacitive behavior and good rate capability. In addition, with increasing scan rates, the redox peaks current intensity areas become larger as the scan sweep increased. Furthermore, due to polarization of the electroactive sample, the anodic peaks shifted enormously towards a positive potential, whereas the cathodic peaks moved towards a negative potential with increasing scan rate because of the low resistance and fast ion and electron transfer rates. The strong peaks can be closely related to the chemical composition and morphology of the electrode material for the reversible faradaic redox reactions and redox reaction corresponding to an alkaline electrolyte according to the equations during the electrochemical measurements.

4 NiCo_2_O_4_ + H_2_O + OH^−^ ↔ NiOOH + 2CoOOH + e^−^

5 2CoOOH + OH^−^ ↔ 2CoO_2_ + H_2_O + e^−^

In addition, galvanic charge–discharge (GCD) measurements were taken to evaluate the specific capacitance of the electroactive material in a 3 M KOH aqueous electrolyte solution between 0–0.5 V (*vs.* Ag/AgCl) at various current densities of 5–10 A g^−1^. The resulting curves were attributed to the faradaic redox process and were close to battery type charge storage, as shown in Fig. 3b. The voltage plateaus in the charge–discharge plots were retained at high scan rates, which indicates the high rate capability of the electrode material. According to eqn (1), the NiCo_2_O_4_ electrode exhibited a specific capacitance of 2791, 2650, 2570, 2530, 2510, and 2490 F g^−1^ at current densities of 5, 6, 7, 8, 8, 9, and 10 A g^−1^, respectively (as shown in Fig. 3c). The specific capacitance was also superior to those of recently reported flexible electrodes with NiCo_2_O_4_ agglomerated particles (351 F g^−1^ at a current density of 1 A g^−1^),^44^ NiCo_2_O_4_ nanoflat-like particles (764 F g^−1^ at the scan rate of 2 mV s^−1^),^45^ NiCo_2_O_4_ flower-like nanostructure (658 F g^−1^ at a current density of 1 A g^−1^),^46^ NiCo_2_O_4_ nanosheets-like (899 F g^−1^ at the current density of 1 A g^−1^),^47^ NiCo_2_O_4_ nanoflakes (1270 F g^−1^ at the current density of 1 A g^−1^),^48^ NiCo_2_O_4_ nanoneedles nanostructure (1427 F g^−1^ at the current density of 8 A g^−1^),^49^ NiCo_2_O_4_ hollow microspheres (764 F g^−1^ at a current density of 2 A g^−1^),^50^ and chain-like NiCo_2_O_4_ nanowires (1284 F g^−1^ at the current density of 2 A g^−1^).^51^

**Fig. 4:**
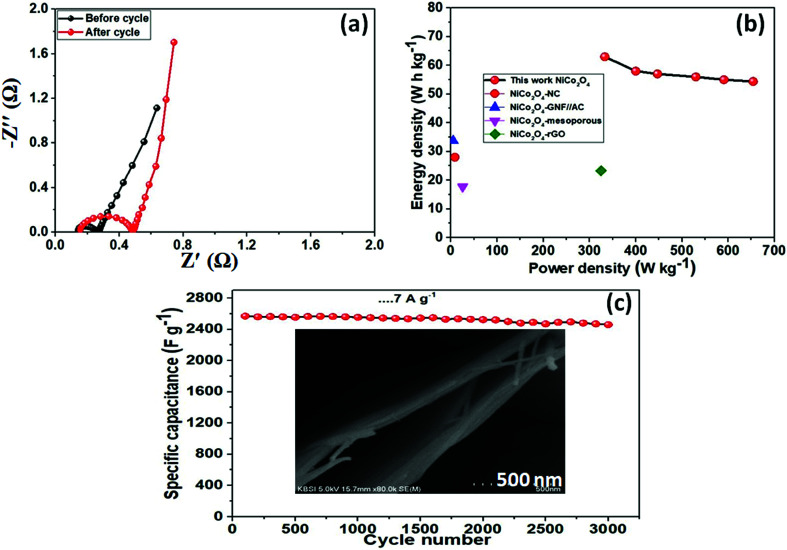
(a) Nyquist plots of the NiCo_2_O_4_ electrode, (b) Ragone plots of the device and (c) cyclic performance of NiCo_2_O_4_ nanoplates during 3000 cycles at a scan rate of 7 A g^−1^.

Fig. 4a shows the Nyquist plot of the device. The electrochemical characteristics of the electrode were examined further by electrochemical impedance spectroscopy (EIS). The Nyquist plots of the electrode were prepared at the open circuit potential over the frequency range, 0.01 Hz to 100 kHz. In the three electrodes, the *R*_s_ value was 0.2 Ω, which indicates the low-frequency range. In addition, the NiCo_2_O_4_ electrode showed an almost vertical line, indicating the diffusion of ions at the interface of the electrode/electrolyte, better conductivity and rapid electron transfer kinetics. Fig. 4b presents Ragone plots of the NiCo_2_O_4_ electrode of the energy and power densities. The device exhibited an excellent energy density of 63.8 W h kg^−1^ at a power density of 333 W kg^−1^, even at a low energy density of 54.4 W h kg^−1^ at a maximum power density of 654 W kg^−1^, which is superior to those reported previously, such as NiCo_2_O_4_-NC (28 W h kg^−1^ and 8.5 W kg^−1^),^52^ NiCo_2_O_4_-GNF//AC (33.8 W h kg^−1^ and 5 W kg^−1^),^53^ NiCo_2_O_4_ mesoporous spinal-like (17.72 W h kg^−1^ and 25.42 W kg^−1^),^54^ and NiCo_2_O_4_-rGO (23.32 W h kg^−1^ and 324.9 W kg^−1^).^55^

The long-term cycling stability and coulombic efficiency of the as-prepared product as an electrode material was examined by repeating the charge–discharge plots. As shown in Fig. 4c, the NiCo_2_O_4_ nanoplate-like structure exhibited excellent long-term electrochemical stability and high rate stability with a very slight decrease to 99.1%, even after 3000 cycles (only 0.9% loss after 3000 cycles) in a three-electrode system at a current density of 7 A g^−1^. This is superior to those of previously reported 3D hierarchical flower-shaped NiCo_2_O_4_ microspheres (93.2% retention after 1000 cycles),^56^ hierarchical spinal NiCo_2_O_4_ nanowires (84.7% retention after 500 cycles),^57^ NiCo_2_O_4_@MnO_2_ nanowire arrays (88% retention after 2000 cycles),^58^ NiCo_2_O_4_@NiO nanowire arrays (93.1% retention after 3000 cycles),^59^ NiCo_2_O_4_@CoMoO_4_ nanowire/nanoplates arrays (74.1% retention after 1000 cycles),^60^ ultrathin porous NiCo_2_O_4_ nanosheet arrays (20% reduced after 3000 cycles),^61^ and NiCo_2_O_4_@NiMoO_4_ nanowires (90.6% retention after 2000 cycles).^62^ TEM showed that the nanohoneycombs still existed in the NiCo_2_O_4_ electrode (inset in Fig. 4c) after long term cycling, suggesting that there was no noticeable change in the morphology of the sample. Such excellent cycling stability of the NiCo_2_O_4_ electrode could be attributed to the following: the unique honeycomb structure, which can alleviate the volume changes during the charge–discharge process to guarantee good stability; gradual penetration of the electrolyte ions into the electroactive material; and reduced volume expansion resulting from the rapid and long-term faradaic reaction.

## Conclusion

NiCo_2_O_4_ with a unique interconnected nanoplate-like structure was fabricated on the surface of the highly conductive nickel foam through a simple chemical bath deposition reaction. The interconnected honeycomb nanostructure electrode can enhance the pathway for electron transport originating from the good electronic conductivity of the nickel foam. In addition, it can reduce the transport distance of ions and enhance electrode–electrolyte contact, which leads to higher material activation, and endow it with a high specific capacitance and excellent long life cycling stability. The NiCo_2_O_4_ nanoplate electrode delivered a high specific capacitance of 2791 F g^−1^ at a current density of 5 A g^−1^ and exhibited a significantly high energy density of 63.8 W h kg^−1^ and high power density of 654 W kg^−1^, highlighting its potential for energy storage applications. The nanoplate structure electrode material for electrochemical capacitors displayed excellent cycling stability of 99.1% retention after 3000 cycles, even at a high current density of 7 A g^−1^. As a result, the NiCo_2_O_4_ electrode provides a path for high-performance supercapacitors because of its low-cost simple chemical bath deposition approach and environmental friendliness.

## Conflicts of interest

There are no conflicts to declare.

## Supplementary Material

RA-009-C8RA09081E-s001
