# Correction: Enhanced electrochemical performance of nanoplate nickel cobaltite (NiCo_2_O_4_) supercapacitor applications

**DOI:** 10.1039/c9ra90096a

**Published:** 2020-01-07

**Authors:** Anil Kumar Yedluri, Hee-Je Kim

**Affiliations:** School of Electrical Engineering, Pusan National University Busandaehak-ro 63beon-gil, Geumjeong-gu Busan 46241 Republic of Korea yedluri.anil@gmail.com +82 51 513 0212 +82 10 3054 8401

## Abstract

Correction for ‘Enhanced electrochemical performance of nanoplate nickel cobaltite (NiCo_2_O_4_) supercapacitor applications’ by Anil Kumar Yedluri *et al.*, *RSC Adv.*, 2019, **9**, 1115–1122.

Attribution to a funding body (BK 21 PLUS) in the published article was incorrect (arising from delays), and the corrected Acknowledgements section should be as follows:

This research was supported by the Basic Research Laboratory through the National Research Foundations of Korea funded by the Ministry of Science, ICT and Future Planning (NRF-2015R1A4A1041584).

The Royal Society of Chemistry apologises for these errors and any consequent inconvenience to authors and readers.

## Supplementary Material

