# Supplementary material for: Enhanced electrochemical performance of nanoplate nickel cobaltite (NiCo2O4) supercapacitor applications
Source: RSC Adv. 2019 Jan 9;9(2):1115–22. doi: 10.1039/c8ra09081e (PMC9059483; doi:10.1039/c8ra09081e)
Supplement: RA-009-C8RA09081E-s001 [file RA-009-C8RA09081E-s001.pdf]

Electronic Supplementary Material (ESI) for RSC Advances.
This journal is © The Royal Society of Chemistry 2018

Supplementary material

Enhanced electrochemical performance of nanoplates nickel cobaltite ($NiCo_2O_4$) supercapacitor applications

Yedluri Anil Kumar* and Hee-Je Kim

[a] School of Electrical Engineering, Pusan National University, Busandaehak-ro 63beon-gil, Geumjeong-gu, Busan, 46241, Rep. of KOREA

*Corresponding Author. Tel: +82 10 3054 8401. Fax: +82 51 513 0212. E-mail: yedluri.anil@gmail.com (A. K. Yedluri); heeje@pusan.ac.kr (H.-J. Kim)

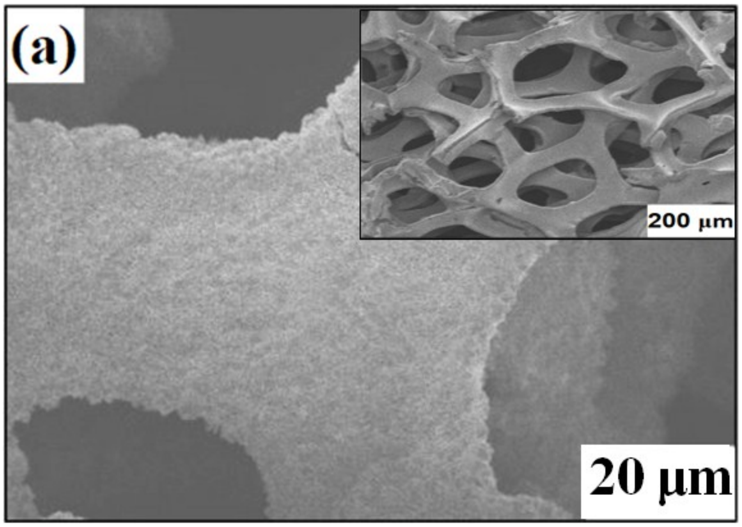

Fig. S1 Low magnification Scanning electron microscope (SEM) images of a) a branch of visible networks of Ni foam surface coated with uniformly covered of $NiCo_2O_4$ nanoplates. Inset: visible networks of Ni foam surface.

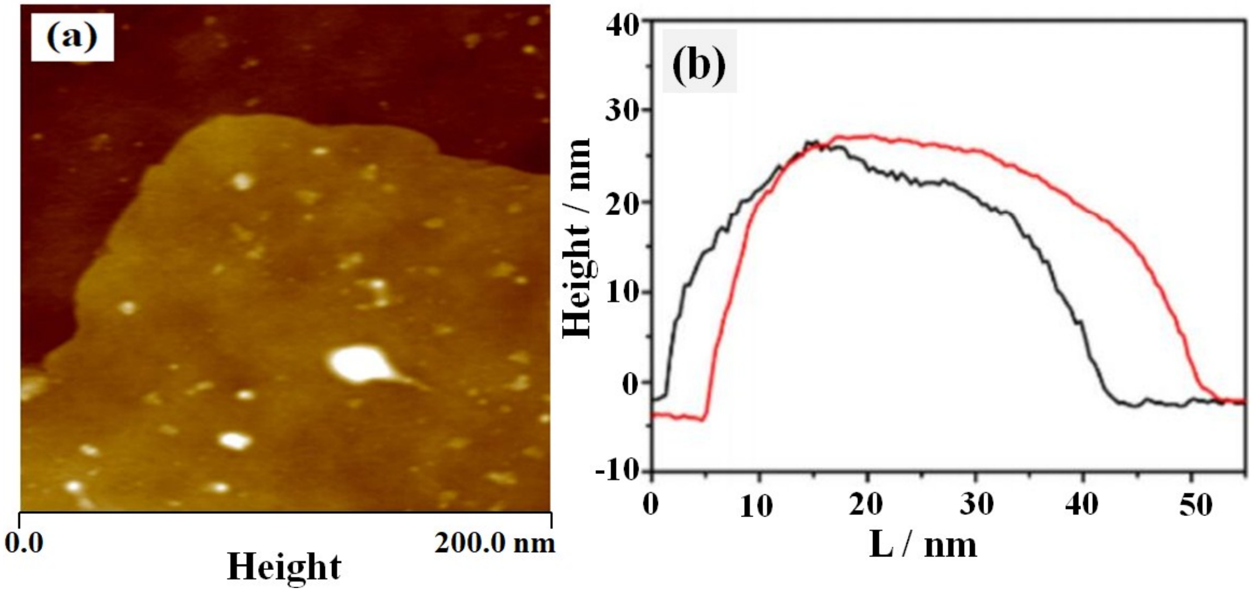

Fig. S2 (a) Representative Atomic Force Microscopy (AFM) analysis of the $NiCo_2O_4$ nanoplate, and (b) corresponding thickness analysis.
